# The Mediating Role of Physical Literacy in the Relationship Between e-Health Literacy and a Sustainable Healthy Lifestyle Among Adolescents

**DOI:** 10.3390/healthcare13151870

**Published:** 2025-07-31

**Authors:** Mehmet Akarsu, Mehmet Güllü, Gül Polat Günata, Aysel Kızılkaya, Savaş Aydın, Ecesu Özcan, Göktuğ Norman, Cihad Onur Kurhan

**Affiliations:** 1 Sports Science Faculty, İnönü University, Malatya 44050, Türkiye; mehmet.gullu@inonu.edu.tr (M.G.); goktug.norman@inonu.edu.tr (G.N.); 2 Health Science Faculty, Malatya Turgut Özal University, Malatya 44050, Türkiye; gul.gunata@ozal.edu.tr; 3 Faculty of Sport Sciences, Fırat University, Elazığ 23000, Türkiye; akizilkaya@firat.edu.tr; 4 Sports Science Faculty, Van Yüzüncü Yıl University, Van 65080, Türkiye; savasaydin@yyu.edu.tr; 5 Sports Science Faculty, Munzur University, Tunceli 62000, Türkiye; ecesuozcan@munzur.edu.tr; 6 Department of Physical Education and Sports, Institute of Health Sciences, İnönü University, Malatya 44050, Türkiye; cihadonurkurhan@gmail.com

**Keywords:** e-health literacy, physical literacy, sustainable healthy lifestyle, adolescents, health behaviors, health education, mediation model

## Abstract

**Background:** It is well-established that, for adolescents to adopt sustainable healthy lifestyle behaviors, not only access to information but also the skills required to translate that information into action are critical. In this field, research that examines the relationship between e-health literacy and sustainable healthy lifestyle behaviors within the context of physical literacy is notably scarce. In this context, the aim of this study is to examine the effect of e-health literacy on a sustainable healthy lifestyle and to evaluate the mediating role of physical literacy in this relationship. **Methods:** A total of 835 adolescents from high schools across Türkiye voluntarily participated in this study. During the data collection process, the e-Health Literacy Scale, the Perceived Physical Literacy Scale, and the Healthy and Sustainable Lifestyle Scale were utilized. Data were analyzed using the JASP (version 0.18.3.0) software. Correlation analysis and structural equation modeling were conducted, and the bootstrap method (n = 5000) was employed for mediation analysis. **Results:** The effect of e-health literacy on a sustainable healthy lifestyle was found to be positive and statistically significant (β = 0.452, *p* < 0.001). Similarly, e-health literacy significantly predicted physical literacy (β = 0.755, *p* < 0.001), and physical literacy significantly predicted a sustainable healthy lifestyle (β = 0.310, *p* < 0.001). The mediating effect was also statistically significant (β = 0.234, *p* < 0.001). The model explained 32% of the variance in healthy lifestyle behaviors. **Conclusions:** The findings indicate that evaluating e-health literacy and physical literacy together provides a holistic approach to fostering sustainable healthy lifestyle habits among adolescents. It is recommended that intervention programs be structured to encompass both areas of competence.

## 1. Introduction

In contemporary public health discourse, the adoption of sustainable healthy lifestyle behaviors has emerged as a central objective of global health policies. However, this goal remains insufficiently realized, particularly among adolescents [1,2]. According to the World Health Organization [3], only 19% of adolescents aged 11 to 17 worldwide meet the recommended levels of physical activity. Similarly low adherence rates have been reported for other health-related behaviors such as healthy eating, adequate sleep, and stress management [4,5]. The failure to acquire these behaviors early in life increases the likelihood of facing risks such as obesity, chronic diseases, mental health problems, and a reduced quality of life in adulthood [6,7]. In this context, the role of digital resources and e-health literacy in promoting sustainable healthy behaviors among adolescents is becoming increasingly critical, surpassing traditional intervention methods. Research indicates that approximately 63% of adolescents utilize digital platforms to seek information in support of a healthy lifestyle [8]. In the context of Türkiye, national surveys indicate that adolescents spend over 3 h per day online on average, yet only a small proportion engage in regular physical activity. According to the Turkish Ministry of Health [9], fewer than 25% of high school students meet the recommended physical activity guidelines, and digital platforms are increasingly used as primary sources of health information.

Although digital platforms offer abundant health-related information, individuals must possess the ability to locate, assess, and effectively use electronic health information to maintain a healthy lifestyle [10,11]. Consistent with this view, previous studies have reported a positive association between adolescents’ e-health literacy and their engagement in healthy behaviors. For example, a study on Turkish high school students revealed a positive relationship between their level of e-health literacy and overall healthy lifestyle behaviors [12]. Similarly, another study reported a significant positive association between adolescents’ e-health literacy and healthy lifestyle behaviors [13]. A recent systematic review and meta-analysis also confirmed that e-health literacy is associated with various health behaviors [14]. In parallel, Dülger and Ayaz-Alkaya [15] found that a health literacy-grounded educational intervention improved adolescents’ dietary behaviors, further reinforcing the idea that digital health skills can significantly support sustainable lifestyle behaviors. These findings suggest that adolescents with high e-health literacy are more likely to engage in behaviors such as regular physical activity, proper nutrition, and preventive health practices. Additionally, e-health literacy may also raise adolescents’ awareness of how environmental choices—such as food sourcing, waste reduction, or eco-friendly consumption—can impact personal and community health, contributing to a broader understanding of a sustainable lifestyle. However, for these behaviors to become sustainable, adolescents must possess the knowledge, skills, and motivational infrastructure that support healthy lifestyle choices. At this point, the concept of physical literacy emerges as a critical variable.

Physical literacy is a multidimensional construct that encompasses the knowledge, skills, motivation, and confidence necessary to support lifelong participation in physical activity [16]. Physical literacy is not limited to physical competence; it also includes affective (motivation and confidence), cognitive (knowledge and understanding), and behavioral (engagement in physical activity) domains [17,18]. These dimensions jointly promote lifelong engagement in health-enhancing behaviors. In fact, physical literacy has been widely recognized as a foundational determinant of sustained participation in physical activity and other health-promoting behaviors across the lifespan [19]. Research has further shown that during adolescence, it serves as a strong predictor of both physical and psychological health outcomes [20]. Accordingly, when digital access to health information afforded by e-health literacy is combined with the behavioral competencies embedded in physical literacy, adolescents are more likely to develop sustainable healthy lifestyle habits. In this context, physical literacy functions as a fundamental bridge that enables individuals to translate health knowledge into action and make lifelong health-related decisions, building on the need to understand the mechanisms underlying these relationships. However, despite the growing evidence, few studies have examined the interplay between e-health literacy and sustainable health behaviors through the mediating lens of physical literacy, particularly in adolescent populations. This study addresses this gap by proposing and empirically testing a conceptual model that integrates these two key literacy domains.

The theoretical justification for considering physical literacy as a mediating variable in the present study is supported by both developmental psychology and behavioral change theories. From a developmental perspective, adolescence is a critical period for identity formation, self-efficacy development, and lifestyle choice consolidation [21]. Physical competencies acquired during this stage influence not only bodily function but also social and cognitive functioning. Moreover, behavioral change theories—such as the Stages of Change Model proposed by Prochaska and DiClemente [22]—suggest that behavior change is not driven by knowledge alone but also requires intrinsic motivation, self-confidence, and practical skills. Physical literacy encompasses precisely these components, thereby acting as a mediator between knowledge acquisition and behavioral transformation.

Despite adolescents’ technological proficiency in accessing online health information, a lack of the practical skills, motivation, and self-efficacy necessary to act on this information may hinder its translation into real-life behaviors. This phenomenon aligns with established health behavior models that emphasize the interaction of cognitive and behavioral competencies. For instance, Nutbeam’s [23] Health Literacy Model conceptualizes health literacy as a personal competency that enables informed health decision-making, while Bandura’s [24] Social Cognitive Theory highlights self-efficacy and behavioral capability as central determinants of behavior change. These frameworks indicate that although individuals with high e-health literacy may be able to access and comprehend quality health information, the extent to which this information leads to behavioral change depends significantly on their level of physical literacy. In other words, e-health literacy not only directly influences healthy lifestyle behaviors but can also indirectly strengthen this influence through physical literacy. Therefore, any intervention aiming to improve adolescents’ health-related behaviors must integrate both e-health and physical literacy as essential and complementary domains.

In this context, the present study aims to examine the relationship between adolescents’ e-health literacy and their sustainable healthy lifestyle. Additionally, it investigates whether physical literacy serves as a mediating factor in this relationship.

### Theoretical Framework

**Figure 1:**
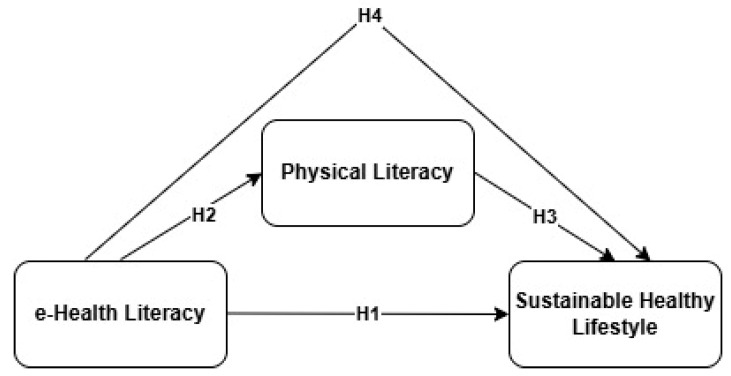
Conceptual framework based on this study’s hypotheses.

Based on the theoretical framework, the following hypotheses were developed and are illustrated in Figure 1:

**H1.**  *Adolescents’ e-health literacy has a positive and significant relationship with their sustainable healthy lifestyle.*

Prior research has consistently shown that adolescents with higher levels of e-health literacy are more likely to adopt healthy behaviors such as physical activity, healthy eating, and preventive practices. Raeside et al. [8] emphasized that adolescents face challenges navigating online health content, but those with higher e-health literacy demonstrate more informed decision-making. Gürkan and Ayar [12] found that higher e-health literacy levels significantly predicted health-promoting behaviors among Turkish high school students. Similarly, Eyimaya et al. [13] reported a positive correlation between adolescents’ e-health literacy and their healthy lifestyle behaviors. Supporting these findings, Kim et al. [14] concluded in their meta-analysis that e-health literacy is consistently associated with a wide range of health-related behaviors across adolescent populations. These collective findings provided the empirical foundation upon which H1 was formulated, linking e-health literacy to sustainable healthy lifestyle behaviors among adolescents.

**H2.**  *Adolescents’ e-health literacy positively predicts their level of physical literacy.*

Recent evidence has shown that e-health literacy contributes not only to informed decision-making but also to physical confidence and competence. Jiang et al. [10] found that Chinese university students with higher e-health literacy levels demonstrated significantly greater physical literacy and were more likely to participate in physical activity. Likewise, Nurjanah et al. [16] reported that adolescents’ physical literacy was positively associated with various aspects of cognitive and emotional engagement fostered by digital health skills. These findings provided the empirical basis for H2, suggesting that e-health literacy may play a predictive role in the development of physical literacy among adolescents.

**H3.**  *Adolescents’ physical literacy positively predicts their sustainable healthy lifestyle.*

Physical literacy encompasses essential components such as motivation, confidence, and knowledge, which are known predictors of sustained health-promoting behaviors including exercise, balanced nutrition, and eco-conscious choices. Whitehead [17] conceptualized physical literacy as a lifelong process crucial to the development of healthy behaviors. Supporting this, UNESCO [18] emphasized that quality physical education fosters physical literacy, which in turn supports lifelong health and well-being. Cairney et al. [19] provided a conceptual model suggesting that physical literacy plays a foundational role in establishing and maintaining active and healthy lifestyles. These perspectives collectively form the theoretical and empirical rationale behind H3, proposing that adolescents with greater physical literacy are more likely to maintain a sustainable healthy lifestyle.

**H4.**  *Physical literacy mediates the relationship between e-health literacy and a sustainable healthy lifestyle among adolescents.*

According to behavioral change models such as Nutbeam’s [23] Health Literacy Model and Bandura’s [24] Social Cognitive Theory, health behavior change requires the interplay of cognitive (e-health literacy) and behavioral (physical literacy) competencies. These frameworks suggest that while e-health literacy provides the necessary knowledge and understanding, physical literacy enables the behavioral execution of that knowledge. Thus, H4 was developed based on the theoretical proposition that physical literacy serves as a mediating mechanism, through which e-health literacy influences sustainable healthy lifestyle behaviors in adolescents.

## 2. Materials and Methods

### 2.1. Study Group

The minimum sample size for this study was calculated using the G*Power software (version 3.1.9.7; University of Düsseldorf, Düsseldorf, Germany). Based on an effect size of 0.03, an alpha level of 0.05, a power (1 − β) of 0.95, and three predictors, a minimum of 577 participants was determined to be required. Data were collected using a convenience sampling method based on accessibility and voluntary participation. Data were obtained from a total of 24 classrooms across three public high schools located in the central district of Malatya, Türkiye. All of these schools are classified as Anatolian high schools. In this context, a total of 850 students were reached, and after excluding 15 participants due to missing data, the analyses were conducted with 835 voluntary participants. Since the data were collected from a single city, the sample does not represent all adolescents in Türkiye. The inclusion criteria were as follows: participants had to be currently enrolled in high school and voluntarily agree to participate after being informed about the purpose and procedures of this study. Participants were required to provide informed consent and complete the face-to-face questionnaire in full. Individuals who declined to participate, provided incomplete responses, attempted to participate multiple times, or whose student status could not be verified were excluded from this study. This study was conducted in accordance with the Declaration of Helsinki and relevant ethical standards. The demographic characteristics of the participants are presented in Table 1.

### 2.2. Research Model

This study was designed using a relational survey model. In the theoretical framework of this study, e-health literacy was positioned as the independent variable, a sustainable healthy lifestyle as the dependent variable, and physical literacy as the mediating variable. Ethical approval for the research was obtained from the Social and Humanities Research and Publication Ethics Committee of Inönü University (Decision No: 10/27).

The model shown in Figure 1 proposes that e-health literacy directly influences sustainable healthy lifestyle (H1), and also indirectly affects it through physical literacy (H2, H3), with physical literacy acting as a mediator (H4).

### 2.3. Data Collection Tools

The sequence of the data collection tools was planned to reduce participants’ cognitive load and to ensure a natural response flow. First, a Demographic Information Form was administered to collect data on basic demographic variables such as age, gender, height, and weight. Following this, the Healthy and Sustainable Lifestyle Scale, which assesses participants’ daily practices, health behaviors, and environmental sensitivity, was administered. Third, the Perceived Physical Literacy Scale, measuring individuals’ knowledge, skills, motivation, and confidence related to physical activity, was applied. In the final phase, the e-Health Literacy Scale was used to evaluate individuals’ ability to access, understand, evaluate, and use health information in digital environments. This sequence was structured to move from general and behavioral content to more cognitive and digital skills, thereby supporting participants’ focus and motivation, and enhancing the reliability of the data collection process.

### 2.4. e-Health Literacy Scale in Adolescents

To evaluate adolescents’ skills in accessing, understanding, evaluating, and using health information in digital environments, the “e-Health Literacy Scale for Adolescents,” developed by Norman and Skinner [25] and adapted into Turkish by Coşkun and Bebiş [26], was used. The scale consists of 8 items and is rated on a 5-point Likert scale (1 = Strongly Disagree, 5 = Strongly Agree). The original study reported a Cronbach’s alpha of 0.780, and in the present study, it was calculated as 0.792. Additionally, confirmatory factor analysis (CFA) was conducted for the e-Health Literacy Scale in Adolescents on this study’s sample, and the results indicated acceptable model fit (χ^2^ = 115.507, Df = 20, CFI = 0.946, TLI = 0.924, NFI = 0.935, IFI = 0.946, RMSEA = 0.076, and SRMR = 0.038), supporting the structural validity of the scale [27]. The scale is designed as a unidimensional tool focusing on general e-health literacy skills rather than dividing them into subdimensions such as functional, interactive, or critical literacy. While the multidimensional nature of e-health literacy is acknowledged in the literature, the unidimensional structure of this scale offers practical advantages in adolescent populations by reducing cognitive burden and improving response reliability.

Example items from the scale include: “I know where to find helpful health resources on the internet.” and “I can evaluate the reliability of online health information.” This scale has been used in multiple Turkish adolescent studies, consistently showing reliable psychometric properties and demonstrating strong cultural alignment with the target population.

### 2.5. Perceived Physical Literacy Scale for Adolescents

To assess adolescents’ physical literacy levels, the Perceived Physical Literacy Scale, developed by Sum et al. [28] and adapted into Turkish by Yılmaz and Kabak [29], was employed. The scale comprises 9 items rated on a 5-point Likert scale (1 = Strongly Disagree, 5 = Strongly Agree). It is structured around three sub-dimensions: (1) Knowledge and Understanding (e.g., “I have sufficient knowledge about physical activity.”), (2) Self-expression and Communication with Others (e.g., “I can share physical activities with others.”), and (3) Sense of Self and Self-confidence (e.g., “I am confident in participating in physical activities.”). The Cronbach’s alpha internal consistency coefficient was reported as 0.900 in the original scale and 0.815 in the present study [29]. The CFA results in the original study indicate an acceptable model fit (χ^2^ = 146.326, Df = 69, CFI = 0.97, TLI = 0.97, and RMSEA = 0.05). Furthermore, the CFA conducted on this study’s sample for the Perceived Physical Literacy Scale for Adolescents indicated acceptable fit indices (χ^2^ = 103.075, Df = 23, CFI = 0.965, TLI = 0.945, NFI = 0.956, IFI = 0.965, RMSEA = 0.065, and SRMR = 0.033), supporting the scale’s structural validity [27]. The scale has also been widely employed in Turkish adolescent research, demonstrating its cultural sensitivity and conceptual adequacy for use in educational and health-related settings in Türkiye.

Although the scale consists of three theoretically meaningful subdimensions, in this study, it was treated as a unidimensional construct. This decision was based on the aim of assessing adolescents’ overall perceived physical literacy level, rather than analyzing each subdomain separately. The three subdimensions are conceptually interconnected and contribute jointly to the holistic development of physical literacy [17]. Previous studies [20,30] also support the treatment of the total score as a valid indicator of general physical literacy, especially in adolescent samples. Thus, the total score was used to reflect participants’ integrated perception of physical literacy in this study.

### 2.6. Healthy and Sustainable Lifestyle Scale

To assess participants’ sustainable healthy lifestyle levels, the Healthy and Sustainable Lifestyle Scale, originally developed by Choi and Feinberg [31] and adapted into Turkish by Gökkaya [32], was used. This scale consists of 28 items rated on a 5-point Likert scale ranging from “1 = Strongly Disagree” to “5 = Strongly Agree.” The scale includes subdimensions such as environmental awareness, healthy nutrition, and physical activity, which align with the multidimensional nature of sustainable healthy lifestyles. The Cronbach’s alpha internal consistency coefficient for the scale was reported as 0.895 [32], while in the present study, it was calculated as 0.855. The CFA results for the original scale indicated a good model fit: χ^2^/Df = 1.895, RMSEA = 0.050, CFI = 0.950, IFI = 0.950, GFI = 0.909, AGFI = 0.903, and RMR = 0.044. In addition, the CFA was conducted on this study’s sample to assess the structural validity of the scale. The results indicated acceptable fit values (χ^2^ = 585.434, Df = 213, CFI = 0.929, TLI = 0.908, NFI = 0.894, IFI = 0.930, RMSEA = 0.046, SRMR = 0.054), confirming the factorial adequacy of the measurement model [27]. Sample items from this scale include: “I engage in regular physical activity.”, “I prefer environmentally friendly products.”, and “I pay attention to healthy eating.” The scale has proven to be both reliable and culturally appropriate in Turkish adolescent populations, supporting its validity and relevance for this study’s context.

Although the scale includes multiple subdimensions such as environmental awareness, healthy nutrition, and physical activity, the total score was used in this study to reflect participants’ overall sustainable healthy lifestyle. This approach aligns with the conceptualization of sustainable lifestyle as a unified behavioral construct encompassing multiple but interrelated health domains [31]. Therefore, the total scale score was employed for analysis rather than examining each subscale separately.

### 2.7. Data Collection Process

Participants were informed in advance about the purpose, scope, and confidentiality principles of the research, and the data collection process was conducted entirely on a voluntary basis. The data were collected between May 5 and May 8, 2025, in a classroom setting at high schools under the direct supervision of the researchers. All data collection took place during regular class hours but outside of examination periods to minimize distraction and stress.

Before participation, participants were presented with an informed consent form, which clearly stated that participation was voluntary, responses would be used solely for scientific purposes, and personal information would remain confidential. Participants were explicitly reminded that there were no right or wrong answers, and they were encouraged to respond honestly, aiming to reduce social desirability bias. The data collection was conducted by trained researchers experienced in adolescent research and field implementation.

The data collection was carried out using a questionnaire form structured in accordance with this study’s objectives. Demographic data were collected first, followed by the application of the measurement tools in a specific sequence. To prevent missing data in the scale forms, responses to all items were required, and the average duration of participation was approximately five minutes per respondent. All data were anonymized and not matched with any personal identifiers. The collected data were securely stored in digital environments for the exclusive purpose of this study. Considering the relatively long structure of the Healthy and Sustainable Lifestyle Scale (28 items), especially when combined with the other tools, specific attention was given to maintaining participant motivation throughout the process. The data collection setting was kept quiet and structured, and clear instructions were provided to reduce fatigue and enhance response accuracy.

### 2.8. Data Analysis

Data analysis was performed using the JASP statistical software (version 0.18.3.0; University of Amsterdam, Amsterdam, The Netherlands). The normality of the data distribution was tested based on skewness and kurtosis values falling within the ±2 range [33,34,35]. The assessments confirmed that the data were normally distributed (Table 2). Accordingly, Pearson correlation analysis was used to examine the relationships among variables.

Before structural equation modeling (SEM), several assumptions were evaluated. Linearity was visually checked through scatterplots. Multicollinearity was assessed via tolerance and variance inflation factor (VIF) values, all of which remained within acceptable limits (VIF < 5, tolerance > 0.20) [36]. Outliers were examined using z-scores; values beyond ±3 were not observed, indicating no extreme outliers in the dataset. Missing data were also addressed: 15 participants were excluded due to incomplete responses, and only fully completed responses were included (n = 835), eliminating the need for further imputation.

In this study, e-health literacy was treated as the independent variable, and sustainable healthy lifestyle was treated as the dependent variable. Physical literacy was considered a mediating variable in this relationship. To test the statistical significance of the mediation effect, a bootstrapping method with 5000 resamples was employed. The resulting 95% confidence intervals were examined to evaluate the significance of indirect effects; the absence of zero within these intervals indicated statistically significant mediation effects [37]. In addition to bootstrapping, z-values were also reported (Table 3) to further support the robustness of the mediation analysis.

## 3. Results

The results obtained within the scope of this study are presented below through tables and figures. In this context, the relationships among e-health literacy, physical literacy, and a sustainable healthy lifestyle are shown in Table 2.

According to the correlation results in Table 2, a significant positive relationship was found between e-health literacy (EHL) and a sustainable healthy lifestyle (SHL) (r = 0.506, *p* < 0.001). A significant positive relationship was also observed between EHL and physical literacy (PL) (r = 0.556, *p* < 0.001). Similarly, a significant positive relationship was found between an SHL and PL (r = 0.495, *p* < 0.001). In this context, the results indicate that there are moderate to strong positive relationships among the variables.

In terms of scale scores, the mean values for all three constructs ranged between 3.19 and 3.71 on a 5-point Likert scale, indicating moderately high levels. Although these values do not represent maximum scores, they suggest that the adolescent participants reported relatively positive tendencies across all domains. The relatively narrow standard deviations (ranging from 0.573 to 0.742) indicate a consistent pattern in responses. A mean score above 3 generally reflects a tendency toward agreement, and thus may be interpreted as a moderate to high level of competence or behavior in the measured domain.

With regard to the distributional assumptions, skewness and kurtosis values for all variables fall within the acceptable ±2 range, suggesting that the data approximate a normal distribution [31,32,33]. Specifically, the skewness values ranged from −0.599 to −0.360 and kurtosis values from 0.545 to 0.839, which are well within thresholds for applying parametric tests such as Pearson correlation and SEM.

Moreover, convergent validity and internal consistency reliability of each latent construct were evaluated using AVE and CR values. All AVE scores exceeded the minimum threshold of 0.50, and all CR values were well above 0.70, indicating that the constructs demonstrate acceptable convergent validity and high internal consistency reliability.

Table 3 presents the findings regarding the mediating role of PL in the relationship between EHL and an SHL. Regarding direct effects, the effect of PL on an SHL was found to be statistically significant (β = 0.310, z = 9.044, 95% CI [0.243, 0.377]), as was the direct effect of EHL on an SHL (β = 0.452, z = 9.717, 95% CI [0.361, 0.544]). Similarly, the direct effect of EHL on PL was also found to be significant (β = 0.755, z = 19.340, 95% CI [0.679, 0.832]). Regarding indirect effects, the effect of EHL on an SHL through PL was found to be statistically significant (β = 0.234, z = 8.192, 95% CI [0.178, 0.290]). As for the total effects, EHL’s total effect on an SHL was significant (β = 0.687, z = 16.935, 95% CI [0.607, 0.766]). The proportion of variance in an SHL explained by EHL and PL was 32%, while the variance in PL explained by EHL was 31%. These findings suggest that physical literacy plays a partial mediating role in the relationship between e-health literacy and a sustainable healthy lifestyle.

According to Cohen’s [38] guidelines, the effect sizes observed in the model are practically meaningful. The direct effect of EHL on PL (β = 0.755) reflects a strong association, while the effect of EHL on an SHL (β = 0.452) is moderate to strong. The indirect effect (β = 0.234) falls within the moderate range, further supporting the significance of the mediating role of PL. These findings indicate the following regarding the hypotheses:

Adolescents’ e-health literacy has a positive and significant relationship with their sustainable healthy lifestyle. This hypothesis is supported by the statistically significant direct effect of EHL on an SHL (β = 0.452, *p* < 0.001).

Adolescents’ e-health literacy positively predicts their level of physical literacy. The strong and significant effect of EHL on PL (β = 0.755, *p* < 0.001) supports this hypothesis.

Adolescents’ physical literacy positively predicts their sustainable healthy lifestyle. This is confirmed by the significant direct effect of PL on an SHL (β = 0.310, *p* < 0.001).

Physical literacy mediates the relationship between e-health literacy and sustainable healthy lifestyle among adolescents. The significant indirect effect (β = 0.234, *p* < 0.001) supports this mediation, indicating that physical literacy plays a partial mediating role in the relationship between EHL and an SHL.

**Figure 2:**
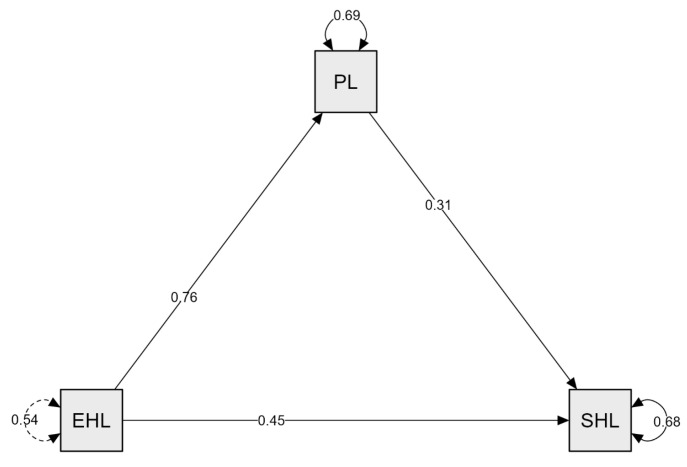
Model created for the mediating role of physical literacy in the relationship between e-health literacy and a sustainable healthy lifestyle.

Figure 2 illustrates the structural equation model examining the mediating role of PL in the relationship between EHL and an SHL. As shown in the model, the direct effect of EHL on PL is both significant and positive (β = 0.76). The direct effect of EHL on an SHL is also significant and positive (β = 0.45). Furthermore, the direct effect of PL on an SHL is significant and positive (β = 0.31).

Following the presentation of the mediation model in Figure 2, several fit indices were examined to assess the model’s adequacy. The model showed a good fit based on commonly accepted thresholds. CFI (0.914), TLI (0.900), IFI (0.915), and RNI (0.914) all exceeded the recommended 0.90 threshold. RMSEA was 0.040, SRMR was 0.050, and GFI was 0.974. These findings indicate that the proposed mediation model adequately explains the relationships among the variables. These findings indicate that the proposed mediation model adequately explains the relationships among the variables.

## 4. Discussion

This study examined the mediating role of physical literacy in the relationship between e-health literacy and a sustainable healthy lifestyle and revealed several important findings. The findings confirmed the hypothesized relationships among the key variables (H1–H4), which were formulated to clarify the structure of this relationship.

First and foremost, the results showed that individuals with higher levels of e-health literacy tend to exhibit more sustainable healthy lifestyle behaviors. This supports H1, underscoring the key role of e-health literacy in transforming health-related knowledge acquired from digital platforms into daily life practices.

Consistent with H2, e-health literacy was also found to positively and significantly predict physical literacy, which in turn directly contributes to the adoption of a sustainable healthy lifestyle. This relationship also supports H3, highlighting the direct influence of physical literacy on health behavior. One of the most notable findings of this study is the significant mediating role of physical literacy in this relationship, providing clear empirical support for H4.

These results suggest that both digital health comprehension and physical competence are crucial and interdependent for promoting healthy lifestyle behaviors. This interpretation is also in line with Bandura’s social cognitive theory, which emphasizes the interaction between knowledge, skills, and behavior in shaping health outcomes.

These findings are consistent with previous studies that emphasize the positive relationship between physical literacy and healthy lifestyle behaviors. For instance, studies by Yalçın and Altun Yılmaz [39], Mayordomo-Pinilla et al. [40], and others reported similar associations among adolescents and young adults. This further supports the mediating role of physical literacy identified in this study.

The correlation results showed moderate and positive associations between e-health literacy, physical literacy, and a sustainable healthy lifestyle, indicating that each of these factors contributes meaningfully to health behavior change.

Building on this, the recent literature increasingly advocates for an integrated approach to e-health and physical literacy, emphasizing their complementary nature rather than treating them in isolation. For example, Jiang et al. [9] and Žnidaršič et al. [41] describe how digital health competence and physical capability are mutually reinforcing. This is particularly relevant for youth, where researchers have urged the enhancement of both digital and physical domains to foster lasting behavior change [42,43,44].

Beyond the individual hypotheses, the overall model demonstrated meaningful explanatory power, supporting the hypothesized relationships. When considered together, e-health literacy and physical literacy explained 32% of the variance in sustainable healthy lifestyle behaviors, which represents a notable but partial contribution given the multifaceted nature of health behavior. Additionally, e-health literacy explained 31% of the variance in physical literacy. These findings underscore the important role of e-health literacy in shaping not only health knowledge but also physical competence and confidence. However, a substantial portion of variance remains unexplained, suggesting that additional factors—such as socioeconomic status, environmental influences, and family context—should be considered in future research to better understand the full range of determinants underlying sustainable healthy lifestyles.

In this respect, the relationship between e-health literacy, physical literacy, and a sustainable healthy lifestyle appears to be more than a superficial correlation. Rather, it may represent an explanatory and potentially causal relationship. An increasing number of studies suggest that both informational competence (e-health literacy) and physical skills and confidence (physical literacy) are key to transforming digital health information into real-life practices [10,41,45]. Especially among young individuals, the ability to transform online health information into behavioral change requires supporting multiple competencies such as movement capability, motivation, and bodily awareness. At this point, physical literacy may serve not only as a mediator but also as a bridging factor connecting digital knowledge to behavior. In this context, the variance ratios identified in this study are significant both theoretically and practically. Models that incorporate both e-health literacy and physical literacy appear to offer an effective framework for understanding and promoting sustainable healthy lifestyle behaviors. This framework may also indirectly support environmental sustainability, as digitally informed adolescents are more likely to understand and adopt health behaviors with ecological benefits, such as sustainable eating or reducing sedentary screen time.

Based on the applied implications of this research, several recommendations can be made. It is not sufficient for educators, health professionals, and policymakers to merely provide information or encourage physical activity. They must also enhance individuals’ physical literacy, namely, their capacity to move, their confidence, and their conscious and sustainable relationship with their bodies. School-based programs, university youth health initiatives, and community-based physical activity interventions should adopt multi-component approaches that integrate both e-health and physical literacy. Especially in intervention programs targeting adolescents and young adults, physical literacy should be considered not only in terms of motor skills but also as a holistic competence encompassing the cognitive and affective skills needed for lifestyle change.

Nevertheless, this study has certain limitations. First, due to its cross-sectional design, causal relationships cannot be definitively established. Future longitudinal or experimental studies are recommended to better examine the temporal dynamics among variables. Second, the data were self-reported, which may introduce bias such as social desirability. Additionally, physical literacy was measured solely based on perception, without incorporating behavioral indicators. Third, the sample consisted only of high school students from a specific region in Türkiye, selected through convenience sampling. Therefore, the generalizability of the findings to other age groups or cultural contexts is limited. Moreover, all participating schools were classified as “Anatolian high schools”, a specific type of public school in Türkiye. Although these schools are widely attended and serve a socioeconomically diverse student population, this sampling frame may still introduce a potential risk of selection bias, which should be considered when interpreting the results. In addition, although procedural measures (e.g., anonymity, voluntary participation) were taken to reduce the likelihood of common method bias, no post-hoc statistical analysis (e.g., Harman’s single-factor test) was conducted, which should be acknowledged as a methodological limitation. Finally, all data were collected using the same method at a single time point, which may carry a potential risk of common method bias. Despite these limitations, this study offers valuable insights and contributes meaningfully to the existing literature.

## 5. Conclusions

In conclusion, the present study demonstrates the necessity of jointly considering two key competence areas—e-health literacy and physical literacy—for promoting sustainable healthy lifestyles. These findings contribute not only theoretically to the academic literature but also offer actionable insights for public health interventions targeting lifestyle changes among adolescents.

Specifically, this study offers a novel theoretical perspective by conceptualizing physical literacy as a behavioral enabler that transforms digital health knowledge into sustained action. This integrated model expands existing health literacy frameworks by bridging informational competence with embodied capability.

Furthermore, this study advances the literature by providing empirical evidence that supports a multidimensional understanding of adolescent health behaviors, highlighting the need to move beyond isolated literacy constructs.

From a methodological standpoint, the use of a mediation model to explore these relationships offers a valuable analytical approach for future health behavior studies. The findings also carry important implications for education and health policy. School-based curricula can be redesigned to incorporate both digital health competencies and movement-related skills, thereby fostering more holistic development among youth.

In light of the digital transformation in health communication—accelerated further by the COVID-19 pandemic—this study highlights the urgency of developing adolescents’ competencies in accessing, interpreting, and applying online health information in physically competent ways.

Future studies are recommended to explore these relationships using longitudinal models and to test them across different cultural contexts. Cross-national comparisons and mixed-method designs may further clarify the causal pathways and context-dependent dynamics of how e-health and physical literacy interact to shape sustainable health outcomes.

## Figures and Tables

**Table 1:** Demographic characteristics of the participants (n = 835).

Title 1	$\bar{x}$	SD
Age (years)	15.83	1.04
Height (cm)	169.44	9.09
Weight (kg)	61.10	13.36
	**n**	**%**
Gender	375 ^1^	44.91
	460 ^2^	55.09

^1^ Male. ^2^ Female.

**Table 2:** Relationships between variables and skewness and kurtosis values.

Variables	EHL	SHL	PL	$\bar{x}$	SD	Skewness	Kurtosis	AVE	CR
EHL ^1^	-	0.506 *	0.556 *	3.507	0.737	−0.584	0.580	0.586	0.892
SHL ^2^	-	-	0.495 *	3.199	0.573	−0.360	0.839	0.591	0.960
PL ^3^	-	-	-	3.710	0.742	−0.599	0.545	0.592	0.928

* *p* < 0.001, ^1^ e-health literacy, ^2^ sustainable healthy lifestyle, ^3^ physical literacy, AVE = average variance extracted, and CR = composite reliability.

**Table 3:** Findings on direct, indirect, and mediating effects.

Direct Effects								%95 Confidence Interval		
					β	Std. Error	z-Value	*p*	Lower	Upper
PL ^3^	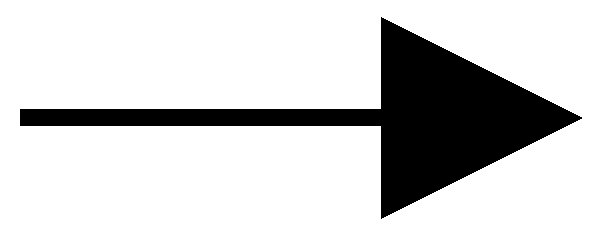	SHL ^2^			0.310	0.034	9.044	<0.001	0.243	0.377
EHL ^1^	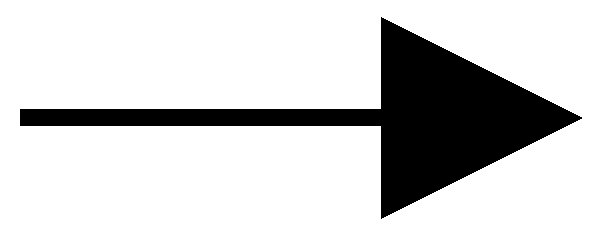	SHL			0.452	0.047	9.717	<0.001	0.361	0.544
EHL	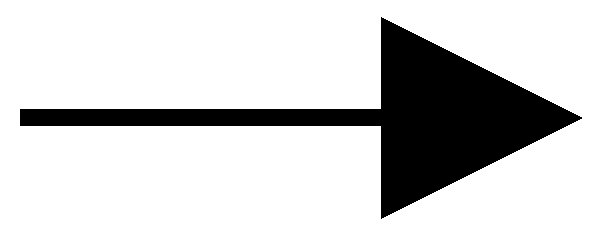	PL			0.755	0.039	19.340	<0.001	0.679	0.832
**Indirect effects**										
EHL	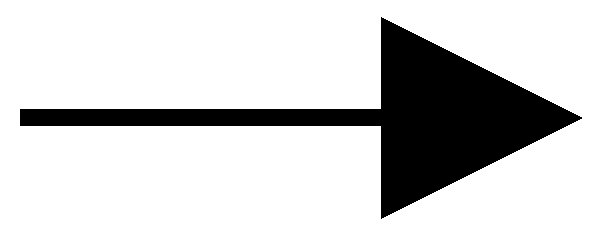	PL	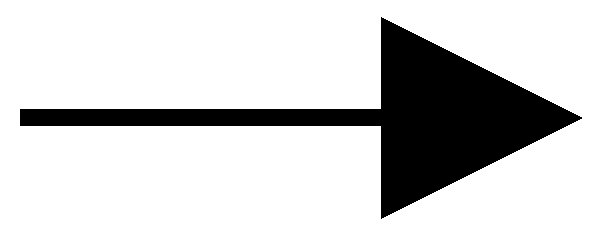	SHL	0.234	0.029	8.192	<0.001	0.178	0.290
**Total effects**										
EHL	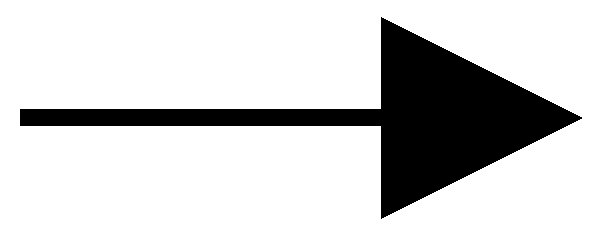	SHL			0.687	0.041	16.935	<0.001	0.607	0.766

^1^ e-health literacy, ^2^ sustainable healthy lifestyle, and ^3^ physical literacy, → = direction of relationship.

## Data Availability

The data presented in this study are available on request from the corresponding author. The data are not publicly available due to ethical and privacy restrictions.

## Abbreviations

The following abbreviations are used in this manuscript:

EHL	e-health literacy
SHL	sustainable healthy lifestyle
PL	physical literacy
$\bar{x}$	mean
SD	standard deviation
CI	confidence interval
β	beta coefficient
z	Z-value
*p*	*p*-value
